# Supplementary material for: MUTATION RATES AND SELECTION ON SYNONYMOUS MUTATIONS IN SARS-COV-2
Source: bioRxiv. 2021 Jan 14:2021.01.14.426705. Preprint. [Version 1] doi: 10.1101/2021.01.14.426705 (PMC7814826; doi:10.1101/2021.01.14.426705)
Supplement: 1 [file NIHPP2021.01.14.426705-supplement-1.pdf]

bioRxiv preprint doi: https://doi.org/10.1101/2021.01.14.426705; this version posted January 14, 2021. The copyright holder for this preprint (which was not certified by peer review) is the author/funder, who has granted bioRxiv a license to display the preprint in perpetuity. It is made available under aCC-BY 4.0 International license.

8 Supplement

8.1 Supplementary Figures

bioRxiv preprint doi: https://doi.org/10.1101/2021.01.14.426705; this version posted January 14, 2021. The copyright holder for this preprint (which was not certified by peer review) is the author/funder, who has granted bioRxiv a license to display the preprint in perpetuity. It is made available under aCC-BY 4.0 International license.

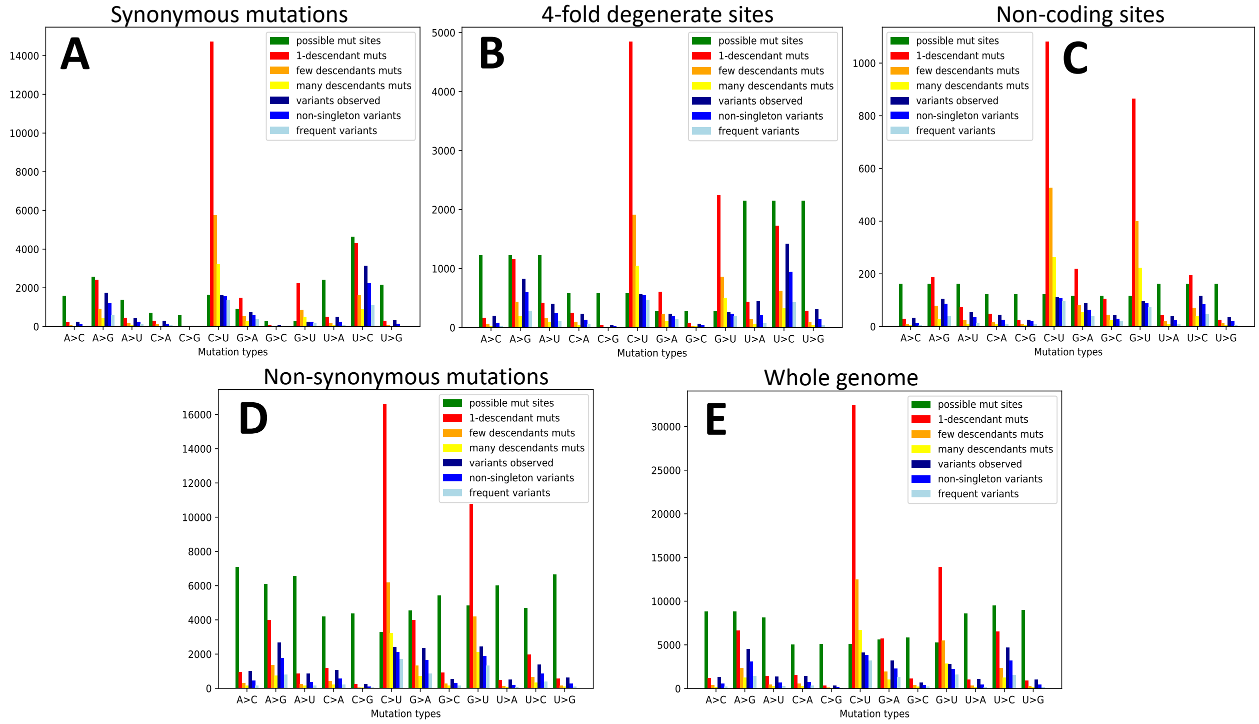

Figure S1: **Numbers of possible mutations, observed mutations, and variable sites in the SARS-CoV-2 genome**. Counts of mutation events for each site class: **A** synonymous sites, **B** 4-fold degenerate sites, **C** non-coding sites, **D** non-synonymous sites, **E** all sites. On the X axis are the 12 distinct types of mutation events, A→C, A→G, etc. In green we show the number of reference sites at which a mutation might have occurred. In red, orange and yellow we show respectively the number of observed mutations with 1 descendant, more than 1 but less than 5 descendants, and more than 4 descendants. In dark blue, blue, and light blue, we show respectively the number of sites with > 0, > 1, and > 4 variants of the given type.

bioRxiv preprint doi: https://doi.org/10.1101/2021.01.14.426705; this version posted January 14, 2021. The copyright holder for this preprint (which was not certified by peer review) is the author/funder, who has granted bioRxiv a license to display the preprint in perpetuity. It is made available under aCC-BY 4.0 International license.

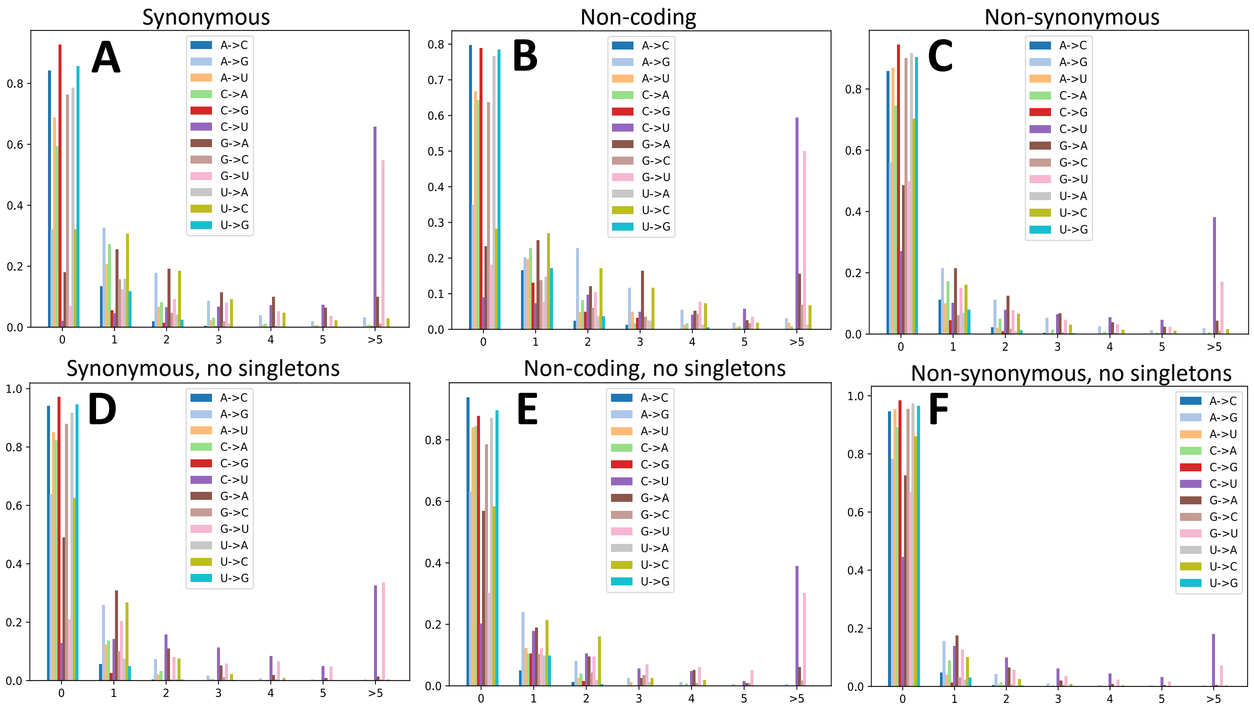

Figure S2: **Re-occurrence of mutation events at the same sites**. Here we show the proportion of sites (Y axis) where a given mutation (color, see legends) appears a certain number of times (X axis) along the phylogeny. **A** synonymous sites; **B** non-coding sites; **C** non-synonymous sites; **D** synonymous sites, but counting only mutation events with more than 1 descendant; **E** non-coding sites, only mutations with more than 1 descendant; **F** non-synonymous sites, only mutations with more than one descendant.

bioRxiv preprint doi: https://doi.org/10.1101/2021.01.14.426705; this version posted January 14, 2021. The copyright holder for this preprint (which was not certified by peer review) is the author/funder, who has granted bioRxiv a license to display the preprint in perpetuity. It is made available under aCC-BY 4.0 International license.

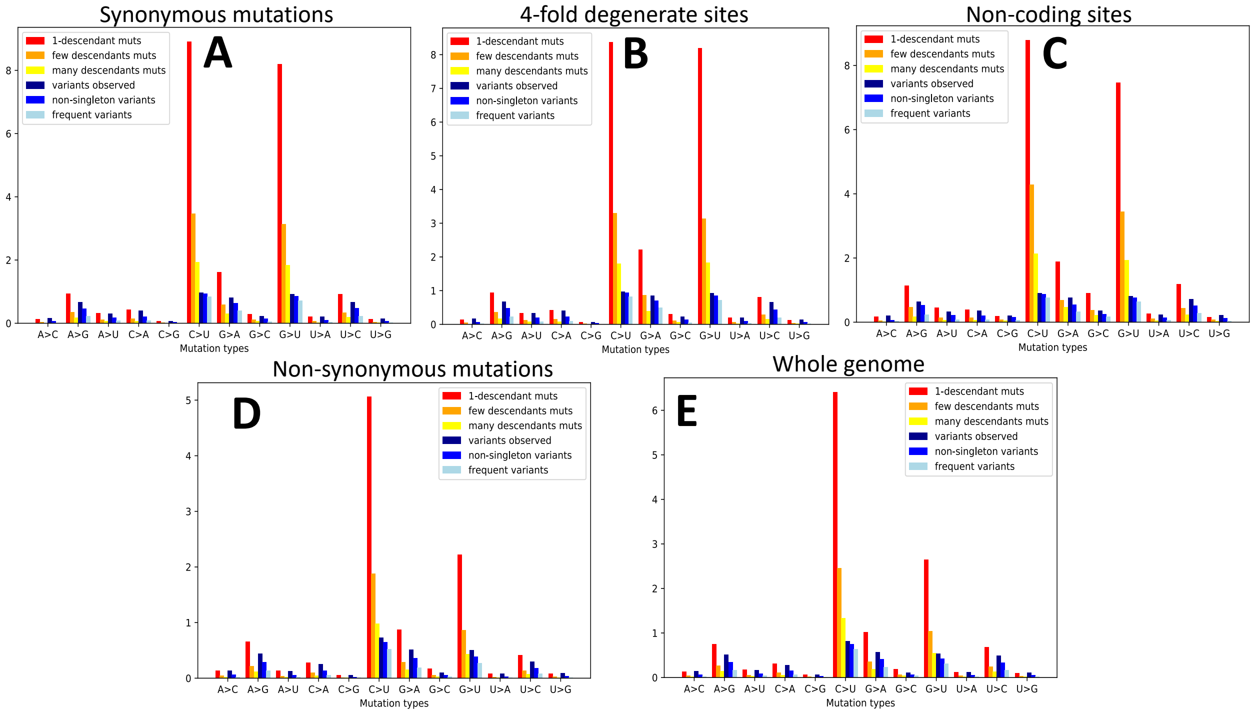

Figure S3: **Mutation rates estimated from mutation counts and variable sites counts**. On the X axis are the 12 distinct types of mutation events, A→C, A→G, etc. On the Y axis are the inferred mutation rates for **A** synonymous sites, **B** 4-fold degenerate sites, **C** non-coding sites, **D** non-synonymous sites, **E** all sites. In red, orange and yellow we show respectively the mutation rates inferred from the numbers of observed mutations with 1 descendant, more than 1 but less than 5 descendant, and more than 4 descendant (and dividing each count by the number of reference sites where such mutations might have happened). In dark blue, blue, and light blue, we show respectively the mutation rates inferred from the numbers of sites with > 0, > 1, and > 4 variants of the given type.

bioRxiv preprint doi: https://doi.org/10.1101/2021.01.14.426705; this version posted January 14, 2021. The copyright holder for this preprint (which was not certified by peer review) is the author/funder, who has granted bioRxiv a license to display the preprint in perpetuity. It is made available under aCC-BY 4.0 International license.

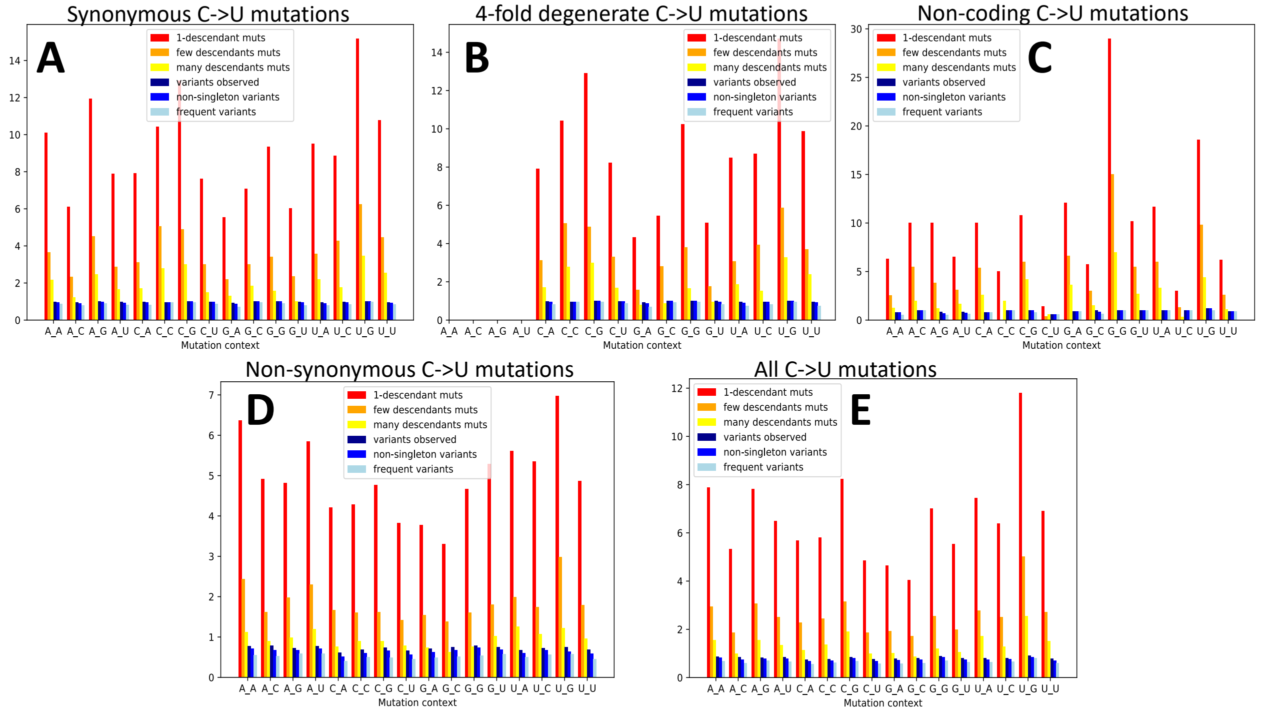

Figure S4: **C→U mutation rates in different base contexts**. C→U mutation rate depending on the previous and next base (5' and 3' base neighbours, shown on the X axis). A_G represents, for example, the trinucleotide ACG and its mutation rate into trinucleotide AUG. Colors are as in legend Figure 3. **A** synonymous sites, **B** 4-fold degenerate sites, **C** non-coding sites, **D** non-synonymous sites, **E** all sites.

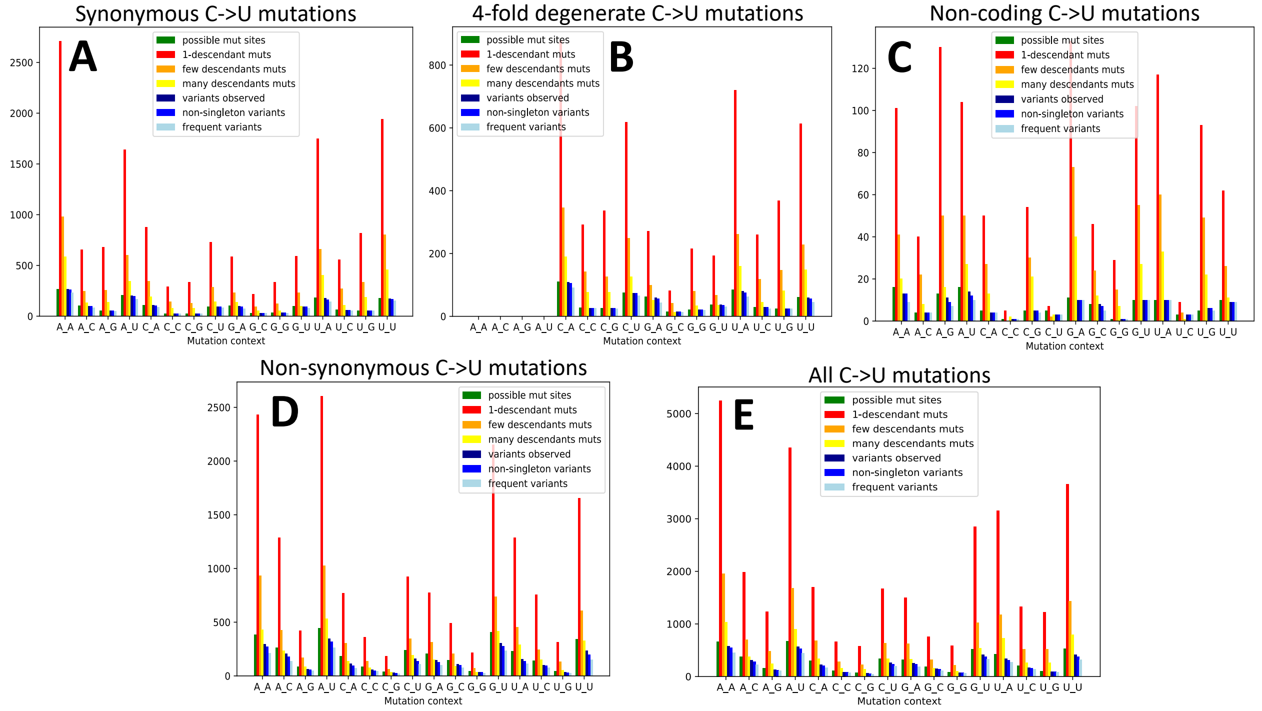

Figure S5: **C→U mutation and mutation possibility counts in different base contexts**. C→U mutation counts depending on the previous and next base (5' and 3' base neighbours, shown on the X axis). A_G represents, for example, the trinucleotide ACG and its mutation counts into trinucleotide AUG. Colors are as in legend Figure 1. **A** synonymous sites, **B** 4-fold degenerate sites, **C** non-coding sites, **D** non-synonymous sites, **E** all sites.

bioRxiv preprint doi: https://doi.org/10.1101/2021.01.14.426705; this version posted January 14, 2021. The copyright holder for this preprint (which was not certified by peer review) is the author/funder, who has granted bioRxiv a license to display the preprint in perpetuity. It is made available under aCC-BY 4.0 International license.

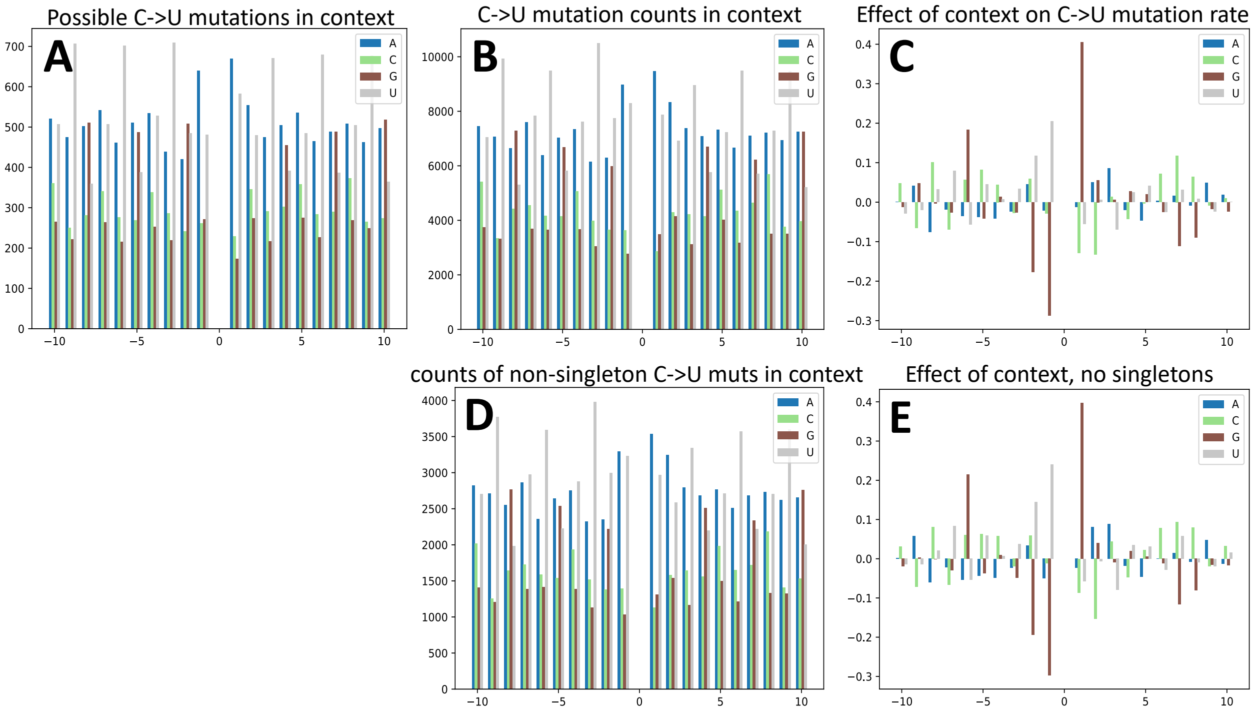

Figure S6: **C→U synonymous mutations and mutation rates in different longer-range base contexts**. Here we consider only synonymous C→U mutations. X axis values represent the distance of the considered base to the one whose mutation rate is considered. Y axis values represent **A** the numbers of possible synonymous mutations with the given context, **B** the numbers of observed synonymous mutations, **D** the numbers of observed non-singleton mutations, **C** the effect on mutation rate that the considered base at the considered position has, **E** same as **C** but without considering mutations with only one descendant. For example, the value for base G at position -1 in plot **C** represents the increase in GC→GU mutation rate vs all other C→U mutation rates; a Y axis value of 0.1 means that the given context increases the background mutation rate by 10%.

bioRxiv preprint doi: https://doi.org/10.1101/2021.01.14.426705; this version posted January 14, 2021. The copyright holder for this preprint (which was not certified by peer review) is the author/funder, who has granted bioRxiv a license to display the preprint in perpetuity. It is made available under aCC-BY 4.0 International license.

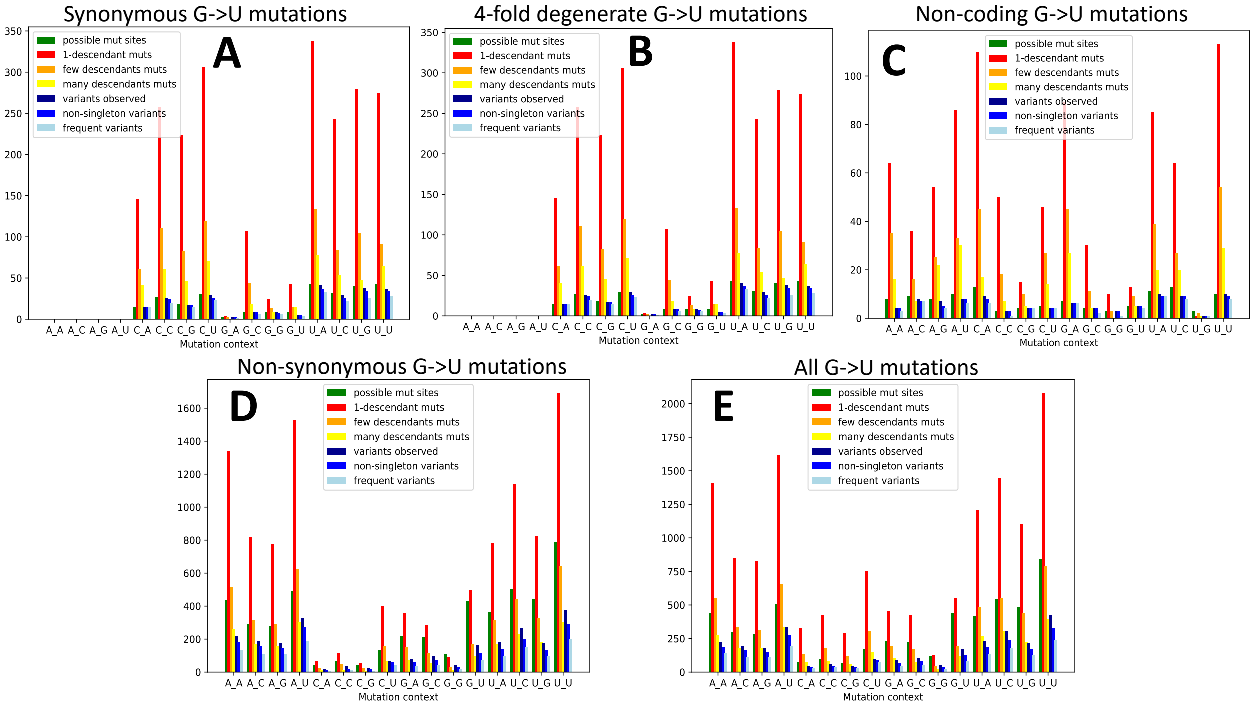

Figure S7: **G→U mutation and mutation possibility counts in different base contexts**. The X axes show the 16 types of mutation contexts for a G→U mutation, for example C_A means the rate of mutation from trinucleotide CGA to trinucleotide CUA. Colors are as in legends and as in Figure 1. **A** synonymous sites, **B** 4-fold degenerate sites, **C** non-coding sites, **D** non-synonymous sites, **E** all sites.

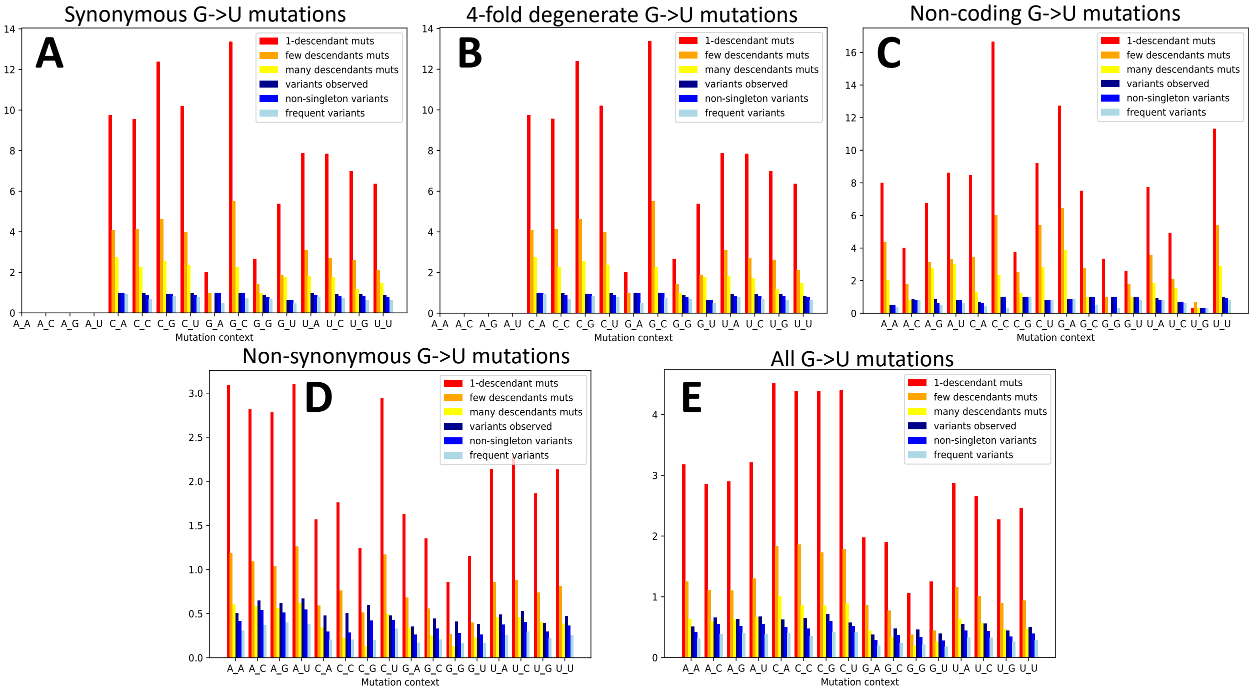

Figure S8: **G→U mutation rates in different base contexts**. G→U mutation rate depending on the previous and next base (5' and 3' base neighbours, shown on the X axis). C_A represents, for example, the trinucleotide CGA and its synonymous mutation rate into trinucleotide CUA. Colors are as in legend Figure 3. **A** synonymous sites, **B** 4-fold degenerate sites, **C** non-coding sites, **D** non-synonymous sites, **E** all sites.

bioRxiv preprint doi: https://doi.org/10.1101/2021.01.14.426705; this version posted January 14, 2021. The copyright holder for this preprint (which was not certified by peer review) is the author/funder, who has granted bioRxiv a license to display the preprint in perpetuity. It is made available under aCC-BY 4.0 International license.

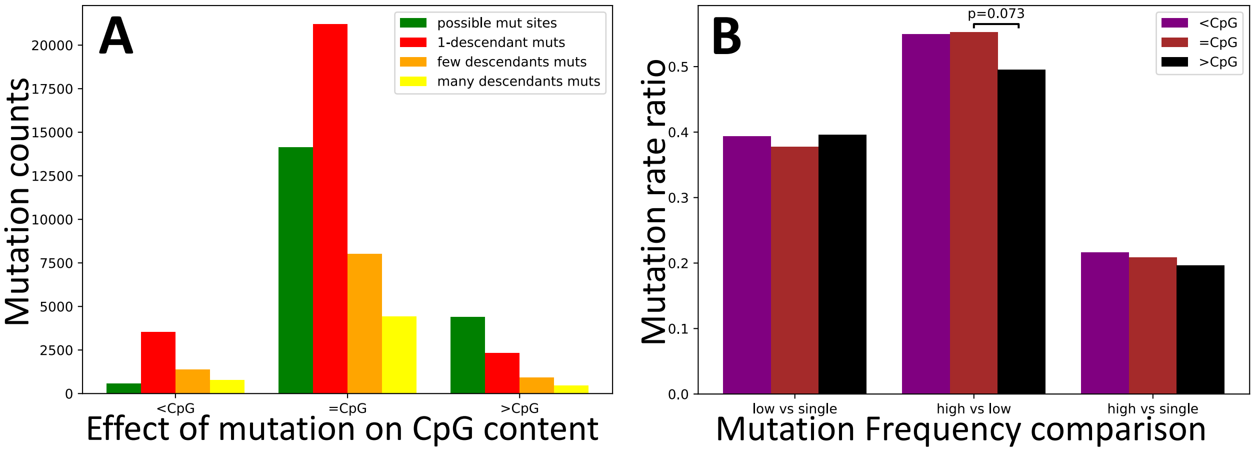

Figure S9: **Test of selection affecting CpG content at synonymous sites.**. Values are the same as in Figure 5, but this time we focus on synonymous mutations that decrease CpG content ("<CpG"), increase it (">CpG"), or leave it unaltered ("=CpG"). Only p-values below 0.1 are shown.

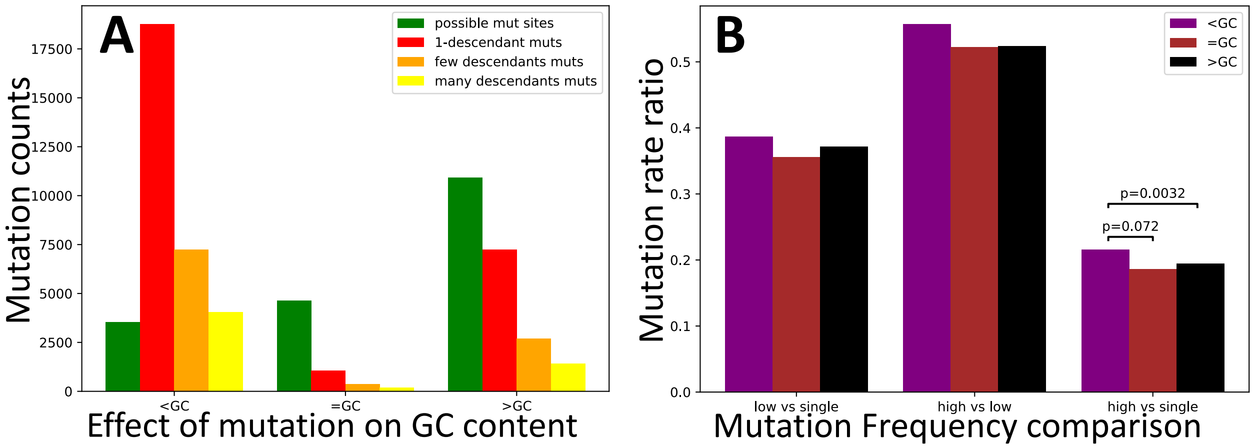

Figure S10: **Test of selection affecting GC content at synonymous sites.**. Values are the same as in Figure 5, but this time we focus on synonymous mutations that decrease GC content ("<GC"), increase it (">GC"), or leave it unaltered ("=GC"). Only p-values below 0.1 are shown.
